# Pheromone cCF10 Enhances Persister Formation in *Enterococcus faecalis* via Transcriptomic Changes

**DOI:** 10.3390/microorganisms14050960

**Published:** 2026-04-24

**Authors:** Jingxue Qian, Xiaobo Yang, Rumeng Li, Man Zhang, Ruolin Hao, Qing He, Lin Xu, Zhiqiang Shen, Jingfeng Wang, Feilong Sun, Zhigang Qiu

**Affiliations:** 1 College of Environmental and Chemical Engineering, Xi’an Polytechnic University, Xi’an 710600, China; 2 Military Medical Sciences Academy, Academy of Military Sciences, Tianjin 300050, China; 3 College of Oceanography and Ecological Science, Shanghai Ocean University, Shanghai 201306, China; 4 The Third Central Clinical College of Tianjin Medical University, Tianjin 300170, China

**Keywords:** *Enterococcus faecalis*, persister cells, pheromone cCF10, pCF10 plasmid, (p)ppGpp, metabolic reprogramming

## Abstract

Bacterial persistence, a non-heritable high-antibiotic-tolerance phenotype, is a key driver of recurrent clinical infections and antibiotic treatment failure. The pheromone-responsive pCF10 plasmid in *Enterococcus faecalis* (*E. faecalis*) mediates antibiotic resistance gene dissemination, but its role in bacterial persister formation remains unclear. This study systematically investigated the regulatory role of pheromone cCF10 in the persister phenotype of pCF10-carrying *E. faecalis* and its underlying molecular mechanisms. We confirmed that cCF10 enhanced persistence against levofloxacin in OG1RF (pCF10), with the persister frequency increasing from 0.291% to 16.466% upon treatment. Transcriptomic analysis revealed that cCF10 activated the (p)ppGpp-mediated stringent response and downregulated the expression of genes associated with energy-intensive pathways, including those involved in DNA repair, protein folding, and respiration. Concurrently, cCF10 enhanced the expression of genes related to biofilm formation and cell lysis resistance and downregulated components of its own sensing and uptake systems. These findings demonstrate that cCF10 induces transcriptional reprogramming associated with increased persister formation in *E. faecalis* carrying the pCF10 plasmid and identify potential targets within the stringent response and associated metabolic pathways for the development of anti-persister strategies.

## 1. Introduction

The emergence of antimicrobial resistance (AMR) poses an existential threat to global public health, with its spread driven by both heritable genetic mechanisms and non-heritable phenotypic adaptations [1,2]. While enzymatic resistance and target modification represent well-characterized heritable strategies, the non-heritable “persister” phenotype also plays a critical role in driving chronic infections and antimicrobial treatment failure [3]. Persisters are defined as a small subpopulation of antibiotic-tolerant bacteria generated via phenotypic heterogeneity. When exposed to lethal concentrations of antibiotics, this subpopulation survives by entering a metabolically quiescent state; once antibiotic stress is relieved, these cells can resuscitate and restore their original antibiotic susceptibility [4,5]. The unique survival characteristics of persisters not only underlie the relapse of chronic and recurrent infectious diseases but also provide a survival reservoir for bacteria under sustained antibiotic pressure. Consequently, they are widely recognized as critical evolutionary intermediates that enable bacteria to acquire high-level heritable resistance [6,7]. Thus, deciphering the molecular mechanisms governing persister formation in major bacterial pathogens represents a key unmet challenge in contemporary anti-infective research.

Microbiological understanding of persister formation has evolved from early stochastic models toward the recognition of programmed physiological stress regulation. Studies on the Gram-negative model organism *Escherichia coli* (*E. coli*) have established the central roles of toxin–antitoxin (TA) modules and the SOS response [8]. For instance, the classic toxin HipA in *E. coli* blocks translation by phosphorylating glutamyl-tRNA synthetase, thereby activating the global metabolic arrest mediated by the alarmone (p)ppGpp [9]; meanwhile, the SOS response has been demonstrated to induce target gene silencing and trigger persistence under fluoroquinolone stress [10]. However, recent studies in Gram-positive pathogens, particularly *Staphylococcus aureus* (*S. aureus*), have revealed a distinct “metabolism-driven” paradigm. Conlon et al. discovered that depletion of intracellular ATP levels serves as the decisive switch triggering the transition to a persistence phenotype, demonstrating that an extreme reduction in core metabolic rate alone suffices to confer multidrug tolerance in bacteria [11]. This concept of energy depletion-induced tolerance has been further corroborated in *E. coli* [12].

*E. faecalis*, a common opportunistic pathogen in clinical infections, has become a key vector for multidrug resistance gene dissemination due to its exceptional environmental adaptability [13]. Clinically, this pathogen is a major cause of infective endocarditis and persistent endodontic infections, where its ability to form biofilms is a defining feature of pathogenesis [14]. Beyond physical protection, *E. faecalis* utilizes TA systems to orchestrate a transition into a dormant persister state. Specifically, the mazEF TA system has been identified as a key regulator of metabolic stasis in clinical isolates, while various plasmid-encoded TA systems are considered ubiquitous elements that ensure genetic stability and stress tolerance in multidrug-resistant *Enterococcus* species [15,16]. Studies have indicated that the stringent response, mediated by synthases such as RelA, reshapes the transcriptome by controlling (p)ppGpp levels, enhancing the bacterium’s stress resistance in host environments [17]. However, the profound biological functions of the cCF10 quorum sensing system—the most characteristic signaling axis of *E. faecalis* centered on the cCF10 signal and its corresponding pCF10 plasmid—in shaping bacterial persister phenotypes remain poorly understood [18].

Recent studies have suggested that in the absence of the pCF10 plasmid, cCF10 may suppress persister formation by regulating energy metabolism pathways [19]; however, this finding presents an apparent discrepancy with the robust persister phenotype we observed in pCF10-harboring clinical *E. faecalis* isolates in this study. Previous logical evidence indicates that plasmid introduction profoundly alters the physiological response pattern of bacteria to the cCF10 signal. According to Breuer et al., plasmid-containing donor cells incur substantial adaptive costs due to the massive expression of polymerizing substances (e.g., PrgB) when responding to cCF10 and inducing the production of conjugation mechanism-related proteins [20]. This short-term, high-intensity protein synthesis and assembly substantially depletes the cells’ limited ATP and amino acid reserves, leading to nutrient diffusion limitations and resulting in significant growth retardation [19,20].

Research by Bhatty et al. further revealed the intrinsic toxicity of this process: inducible *prgB* expression induces envelope stress and even triggers partial cell lysis, compelling bacteria to mobilize negative regulators like PrgU for post-transcriptional “risk management” to maintain intracellular homeostasis [21,22]. This signal-driven metabolic burden aligns intrinsically with the “metabolic collapse-induced dormancy” theory.

Therefore, we hypothesized that the root of this discrepancy lies in the strict genetic background dependence of cCF10’s regulatory function: the aforementioned study was conducted exclusively in plasmid-free *E. faecalis* strains, where cCF10 can only act on chromosomal targets related to energy metabolism. In contrast, in pCF10-carrying strains, the plasmid-encoded high-affinity cCF10 receptor PrgX acts as the dominant intracellular target of cCF10 [20,23], which fundamentally rewires the downstream signaling output of cCF10.

This plasmid-dependent functional rewiring of cCF10 has not been addressed in previous studies, and the mechanism by which it modulates persister formation remains unclear. Accordingly, this study investigates the regulatory effect of cCF10 on persister formation in pCF10-carrying *E. faecalis* OG1RF, characterizes the transcriptomic changes in both exponential-phase cells and persister cells, and identifies core pathways mediating cCF10-enhanced persistence, aiming to reveal the underlying molecular mechanisms.

## 2. Materials and Methods

### 2.1. Strains and Culture Conditions

The bacteria used in this study were all derived from *E. faecalis* OG1RF (ATCC47077). The pheromone-responsive pCF10 plasmid [18,24,25], which carries a tetracycline resistance gene, was a gift from Professor Gary Dunny of the University of Minnesota, USA. The bacteria were grown at 37 °C in Brain Heart Infusion (BHI) medium (Coolaber Science and Technology, Beijing, China) supplemented with 10 mg/L Tc.

### 2.2. Establishment of the E. faecalis Persistence Screening Model

Levofloxacin (LVFX) is widely used as a fluoroquinolone antibiotic to study the persister formation of *E. faecalis* and other Gram-positive pathogens.

First, log-phase *E. faecalis* OG1RF (pCF10) was inoculated at a 1:300 ratio into BHI medium supplemented with 10 mg/L Tc and incubated for 4 h. The culture was then exposed to different concentrations of levofloxacin hydrochloride (LVFX). An untreated group without LVFX exposure was set up as the control. Samples were collected at designated time points, and cultures were serially diluted in phosphate-buffered saline (PBS). Appropriately diluted samples were then plated onto BHI agar to enumerate viable bacteria (CFU/mL). All experiments in this section were performed with at least 3 independent biological replicates, and each biological replicate included 3 technical replicates.

### 2.3. Experiment on the Effect of cCF10 on the Persistence Formation of E. faecalis OG1RF (pCF10)

First, log-phase *E. faecalis* OG1RF (pCF10) was inoculated at a 1:300 ratio into BHI medium supplemented with 10 mg/L Tc. After incubation at 37 °C for 1 h, different concentrations of pheromone cCF10 (0.01 μg/L, 0.1 μg/L, 0.5 μg/L, 1 μg/L, 5 μg/L, 10 μg/L, 15 μg/L, 20 μg/L, 25 μg/L, and 30 μg/L) were added, and incubation was continued for 3 h (the control group received no pheromones). The mixture was then centrifuged at 8000 rpm for 3 min to collect the bacteria. The collected bacteria were resuspended in BHI medium, and LVFX was added to a final concentration of 20 mg/L. The mixtures were incubated for 10 h at 37 °C to kill the sensitive cells. The surviving bacteria collected represented the persisters. The persister frequency *f* was calculated using the following formula:

$$f=\frac{N_{2}}{N_{1}}$$

where N_2_ is the bacterial count (CFU/mL) in the culture after 10 h of antibiotic treatment, and N_1_ is the bacterial count (CFU/mL) in the culture prior to antibiotic treatment. All experiments in this section were performed with at least 3 independent biological replicates, and each biological replicate included 3 technical replicates.

The pheromone cCF10 (amino acid sequence: LVTLVFV) [26] used in this study was synthesized by GenScript Biotech Corporation (Nanjing, China). The purchased cCF10 pheromone was dissolved in dimethyl sulfoxide (Shanghai Meilin Biochemical Technology Co., Ltd., Shanghai, China) and stored at −20 °C protected from light.

### 2.4. Experiment on the Effect of cCF10 on the Growth of E. faecalis

Overnight cultures of *E. faecalis* OG1RF and *E. faecalis* OG1RF (pCF10) were each diluted to 1 × 10^5^ CFU/mL in BHI broth. Then, different concentrations of cCF10 (0.1 μg/L, 10 μg/L, and 30 μg/L) were added to the bacterial cultures (the control group received no pheromones). The mixed cultures were transferred to a 100-well plate (200 μL per well) and the OD600 was measured using an automated growth curve analyzer (Bioscreen C, Helsinki, Finland).

### 2.5. Preparation of Scanning Electron Microscopy (SEM) Samples

First, *E. faecalis* OG1RF (pCF10) was inoculated into BHI medium supplemented with 10 mg/L Tc. After 4 h of incubation at 37 °C to reach the logarithmic growth phase, the bacterial culture was treated with LVFX at a final concentration of 20 mg/L. 1 mL of culture medium containing normal bacteria and persisters were collected after 10 h of antibiotic treatment and centrifuged at 6000 rpm for 5 min. After the supernatant was removed, cells were resuspended in pre-chilled 2.5% glutaraldehyde fixative and fixed at 4 °C for 24 h. The fixative was then removed by centrifugation at 6000 rpm for 5 min. Samples were washed three times with PBS before dehydration via graded ethanol series. Dehydration was performed sequentially using 30%, 50%, 70%, 80%, 90%, 95%, and 100% ethanol solutions. Anhydrous ethanol was used for dehydration twice, with 10 min of standing each time. Finally, the pellets were freeze-dried for 24 h using a lyophilizer. Morphological structures of bacteria were observed using SEM (Zeiss Sigma300, Oberkochen, Germany).

### 2.6. Transcriptome Sequencing

First, normal bacteria, persisters, and persisters treated with cCF10 were collected from *E. faecalis* OG1RF (pCF10) as previously described, with 3 independent biological replicates set for each experimental group. Bacterial pellets were immediately frozen in liquid nitrogen for 15 min and stored at −80 °C until RNA extraction.

RNA library construction and high-throughput sequencing were performed by Shanghai Personal Biotechnology Co., Ltd. (Shanghai, China). Sequencing was performed on the Illumina NovaSeq 6000 platform with a paired-end 150 bp (PE150) read length, with a minimum of 2 Gb raw sequencing data generated per sample. Raw data were filtered using Fastp (0.22.0) software, with a quality control threshold of Q30 ≥ 93% for all clean data used in subsequent analysis. Clean reads were mapped to the *E. faecalis* OG1RF reference genome using Bowtie2 (2.4.1) software, with total mapping rates ranging from 85.92% to 97.36% and unique mapping rates above 99.66% across all samples, ensuring sufficient genome coverage and data reliability. The detailed sequencing data processing and analysis workflow is provided in Supplementary Text S1.

### 2.7. Total RNA Extraction and Real-Time Fluorescent Quantitative Reverse Transcription Polymerase Chain Reaction Analysis

First, collected bacterial samples were immediately transferred to ice for cooling. Total RNA was extracted according to the instructions of the High Purity RNA Extraction Kit (Zhongshitongchuang, Tianjin, China). Total RNA concentration was measured using NanoDrop ONE™ (Thermo Fisher Scientific, Waltham, MA, USA). Subsequently, total RNA was reverse-transcribed to cDNA using a heat-resistant first-strand cDNA synthesis kit (ZhongShitongchuang, Tianjin, China) with random primers. qRT-PCR analysis was performed using PowerUp SYBR Master Mix (ThermoFisher, USA) and the CFX96 real-time System (Bio-Rad Laboratories Inc., Hercules, CA, USA). mRNA levels of target genes were determined by means of absolute quantification using standard curves generated for each gene. The standard curves of the target gene are detailed in Supplementary Figure S1. The qRT-PCR reaction system had a total volume of 20 μL, comprising 10 μL of PowerUp SYBR Master Mix (A25742, Massachusetts Applied Biosystems, Waltham, MA, USA), 2 μL of primers, 1 μL of cDNA, and 7 μL of nuclease-free water. The *16S rRNA* gene was used as the endogenous reference gene to normalize the expression levels of target genes.

The primer sequences for qRT-PCR analysis are provided in Supplementary Table S1 and were designed with the assistance of DNAStar (11.1). All qRT-PCR experiments were performed with 3 independent biological replicates, and each biological replicate included 3 technical replicates.

### 2.8. Statistical Analysis

All data were statistically analyzed using GraphPad Prism 9.5 software. All experiments were performed with at least three independent biological replicates, and data are presented as mean ± standard deviation (SD). Unpaired *t*-tests were performed for comparisons between two groups, while one-way analysis of variance (ANOVA) was used for comparisons among multiple groups. Prior to statistical analysis, data normality was assessed using the Shapiro–Wilk test, and homogeneity of variance was evaluated using Levene’s test. Statistical significance was indicated by asterisks: * *p* < 0.05; ** *p* < 0.01.

## 3. Results

### 3.1. The Pheromone cCF10 Promoted the Formation of Persister Cells in E. faecalis OG1RF (pCF10)

**Figure 1:**
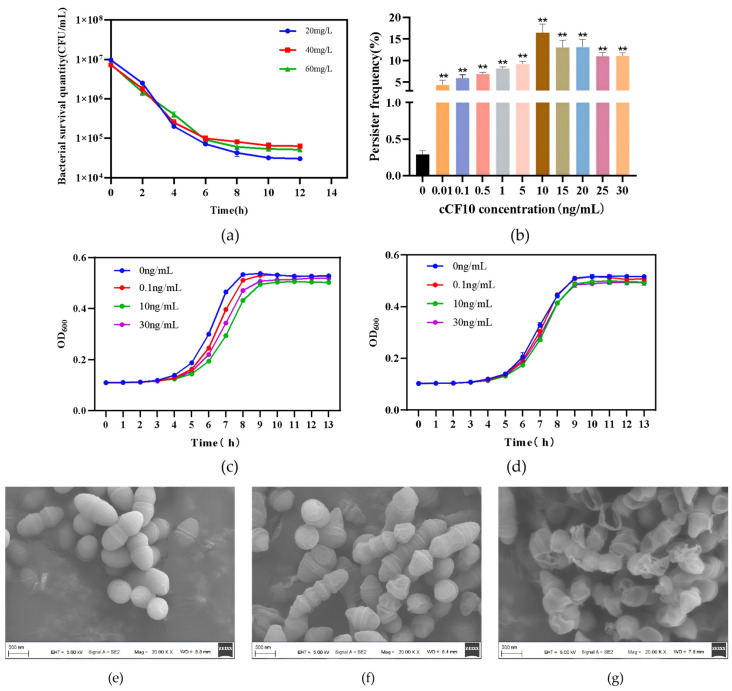
Phenotypic verification of cCF10-induced persister formation in *E. faecalis* OG1RF (pCF10). (**a**) Biphasic time–kill curves of the strain treated with LVFX. (**b**) Effect of gradient concentrations of cCF10 on the persister frequency of the strain. (**c**) Growth curves of *E. faecalis* OG1RF (pCF10) treated with different concentrations of cCF10. (**d**) Growth curves of plasmid-free wild-type *E. faecalis* OG1RF treated with different concentrations of cCF10. (**e**) Scanning electron microscopy (SEM) observation of the morphological characteristics of normally growing *E. faecalis* OG1RF (pCF10). (**f**) SEM observation of the morphological characteristics of *E. faecalis* OG1RF (pCF10) persister cells. (**g**) SEM observation of the morphological characteristics of cCF10-treated *E. faecalis* OG1RF (pCF10) persister cells. The results represent the mean ± SD of three biological replicates. Significant differences between groups are presented with ** *p* < 0.01.

To investigate the persister characteristics of *E. faecalis* OG1RF (pCF10), we first established a persister cell screening model for this strain in this study. Based on the minimum inhibitory concentration (MIC) of LVFX against *E. faecalis* OG1RF (pCF10) of 2 mg/L (Supplementary Figure S2), we conducted biphasic time–kill curve experiments using LVFX at 10× MIC. The results showed that the strain exhibited a typical biphasic time–kill curve dynamic process under LVFX exposure: at the onset of antibiotic treatment, the number of viable bacteria decreased rapidly; as the exposure time increased, the bacterial killing rate slowed, and the bacteria entered a survival plateau after 10 h of exposure. When the LVFX concentration was further increased to 40 mg/L and 60 mg/L, no further significant reduction in the number of surviving bacteria was observed (Figure 1a). To confirm the identity of persister cells, we performed regrowth assays and MIC re-determination of the surviving bacteria after antibiotic removal; the results confirmed that the surviving bacteria resuscitated in antibiotic-free medium and restored the same LVFX susceptibility as the original parental strain (Supplementary Figure S3). These findings indicated that the strain formed persisters under high antibiotic pressure, and thus, the *E. faecalis* OG1RF (pCF10) persister research model was successfully established [27,28,29].

Based on this model, we pretreated the strains with different concentrations of cCF10 for 3 h before persister screening and then calculated the persister frequency of bacteria in each group. Our results indicated that all tested concentrations of cCF10 increased the persistence rate of *E. faecalis*, and the increase in persister frequency exhibited a cCF10 concentration-dependent pattern. The persister frequency in the control group was only 0.291%, while in the 10 μg/L cCF10 treatment group, the persister frequency reached a peak of 16.466%; treatment groups with higher concentrations of cCF10 still maintained a significant persister-promoting effect. This finding indicated that cCF10 promoted persister formation in *E. faecalis* OG1RF (pCF10) (Figure 1b).

To rule out the possibility that differences in bacterial growth induced by cCF10 treatment might interfere with the persister frequency results, and to verify the specificity of cCF10’s biological effects, we examined the growth curves of *E. faecalis* OG1RF (pCF10) and the wild-type OG1RF strain after treatment with different concentrations of cCF10. The results showed that cCF10 concentration-dependently slowed the logarithmic growth rate of the OG1RF (pCF10) strain; however, no decrease in total viable cell count was observed in any of the cCF10-treated groups. This suggested that cCF10 did not directly reduce bacterial viability, but instead slowed bacterial proliferation via physiological regulation rather than affecting bacterial survival via cytotoxic effects (Figure 1c). Meanwhile, cCF10 treatment had no significant effect on the growth rate of the OG1RF wild-type strain lacking the pCF10 plasmid (Figure 1d). The results confirmed that the regulatory effect of cCF10 on bacterial growth and the promoting effect on the formation of persisters were specific biological effects mediated by the pheromone-responsive system encoded by the pCF10 plasmid. Furthermore, the extent of cCF10-mediated downregulation of bacterial growth rate was highly consistent with the concentration-dependent trend of its persistence-promoting effect, suggesting that cCF10-induced downregulation of growth metabolism is a crucial physiological basis for its promotion of *E. faecalis* OG1RF (pCF10) persister formation.

In this study, we further performed SEM to characterize the morphological features of the surviving bacterial population after antibiotic exposure. SEM results showed that normally growing *E. faecalis* OG1RF (pCF10) cells were nearly spherical, with a plump and regular shape, and were distributed uniformly (Figure 1e). Surviving bacteria recovered after LVFX treatment exhibited cell shrinkage and structural deformation (Figure 1f). Bacteria pretreated with 10 μg/L cCF10 prior to antibiotic exposure showed more pronounced shrinkage and deformation, with visible extracellular matrix adherent to cell surfaces (Figure 1g). These ultrastructural alterations were indicative of a stressed, antibiotic-tolerant state and aligned with the phenotypic changes expected in persister cells.

### 3.2. cCF10 Regulated the Staged Molecular Mechanism of Persistence Formation in OG1RF (pCF10)

#### 3.2.1. cCF10 Mediated Pre-Adaptive Programming of OG1RF (pCF10) Persistence Formation via Multi-Pathway Transcriptional Regulation

For transcriptome sequencing, persister cells were isolated using the exact same standardized protocol as the persister screening model. All samples underwent identical processing for collection, RNA extraction, library construction, and sequencing to ensure comparability across groups.

**Figure 2:**
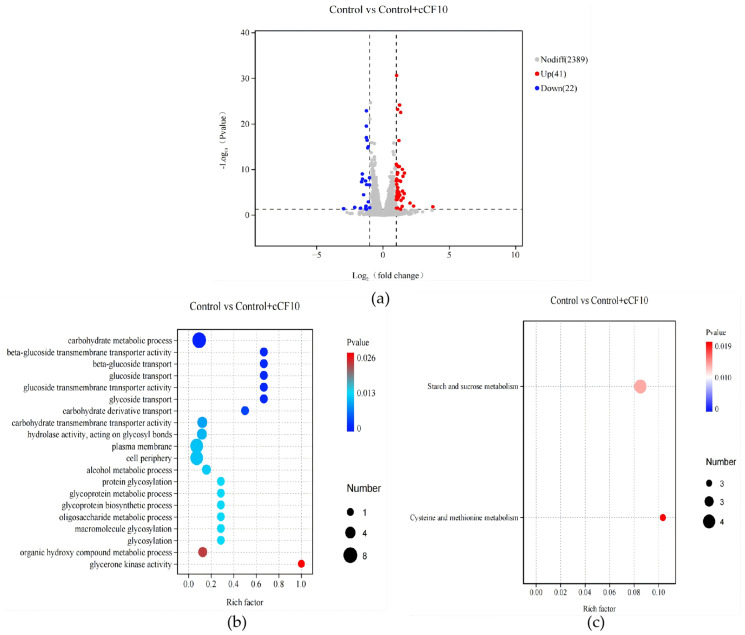

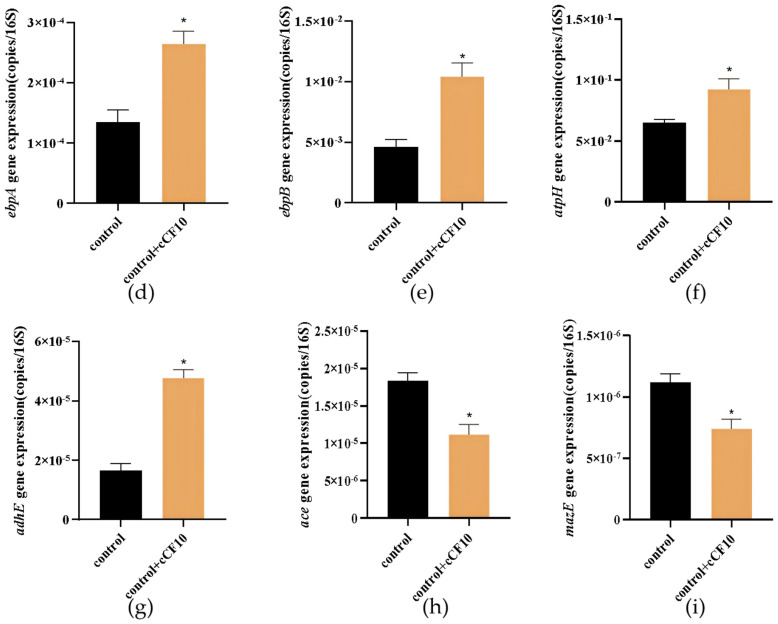
Transcriptomic profiling of cCF10-treated log-phase *E. faecalis* OG1RF (pCF10) and qRT-PCR validation of key genes. (**a**) Volcano plot of differentially expressed genes (DEGs) between the control group and cCF10-treated group in exponential-phase bacteria. (**b**) Bubble plot of GO functional enrichment analysis for DEGs. (**c**) Bubble plot of KEGG pathway enrichment analysis for DEGs. (**d**–**i**) Relative expression levels of key genes related to persister formation. The results represent the mean ± SD of three biological replicates. Significant differences between groups are presented with * *p* < 0.05.

Transcriptomic sequencing of OG1RF (pCF10) in the exponential growth phase was performed, and volcano plot analysis of differentially expressed genes (DEGs) identified a total of 63 significant DEGs. This set of DEGs reflected a targeted transcriptional response to cCF10 in actively growing cells. Differentially expressed genes (DEGs) were defined using a significance threshold of |log_2_ (fold change)| > 1 and *p*-value < 0.05. GO and KEGG enrichment analysis showed that these DEGs were primarily involved in carbohydrate metabolism, transmembrane transport, cell adhesion, and TA system regulation (Figure 2b,c).

Gene expression analysis revealed that cCF10 treatment increased the expression of the fimbriae subunit-encoding genes *ebpA* and *ebpB* by 1.96-fold and 2.24-fold, respectively, while downregulating the transcriptional level of the collagen-binding protein-encoding gene *ace* (Figure 2d,e,h). These expression changes suggest a potential role for cCF10 in modulating bacterial adhesion and aggregation phenotypes, which may contribute to biofilm-associated persistence. cCF10 also upregulated the ethanol dehydrogenase gene *adhE* and ATP synthase subunit gene *atpH* by 1.41-fold and 2.89-fold, respectively (Figure 2f,g). These transcriptional changes in energy metabolism-related genes align with a potential shift in fermentative pathway utilization, which may support a transition to a metabolically quiescent state. Simultaneously, cCF10 downregulated the expression of the gene *mazE* (Figure 2i), the gene encoding the antitoxin component of the MazEF TA system, which may relieve the antitoxin’s inhibitory effect on its cognate toxin, trigger the activation of bacterial dormancy-related regulatory programs, and thus contribute to the molecular basis of persister formation.

#### 3.2.2. Stability and Antibiotic Tolerance of cCF10-Enhanced Persister Cells

**Figure 3:**
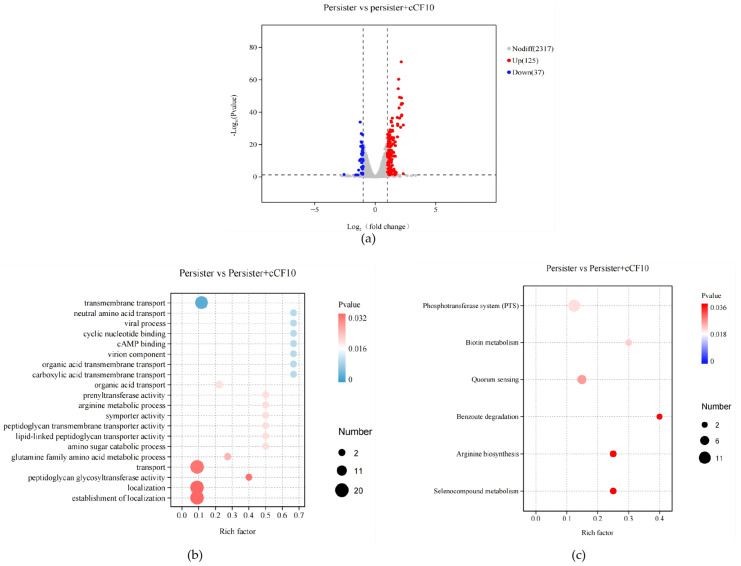

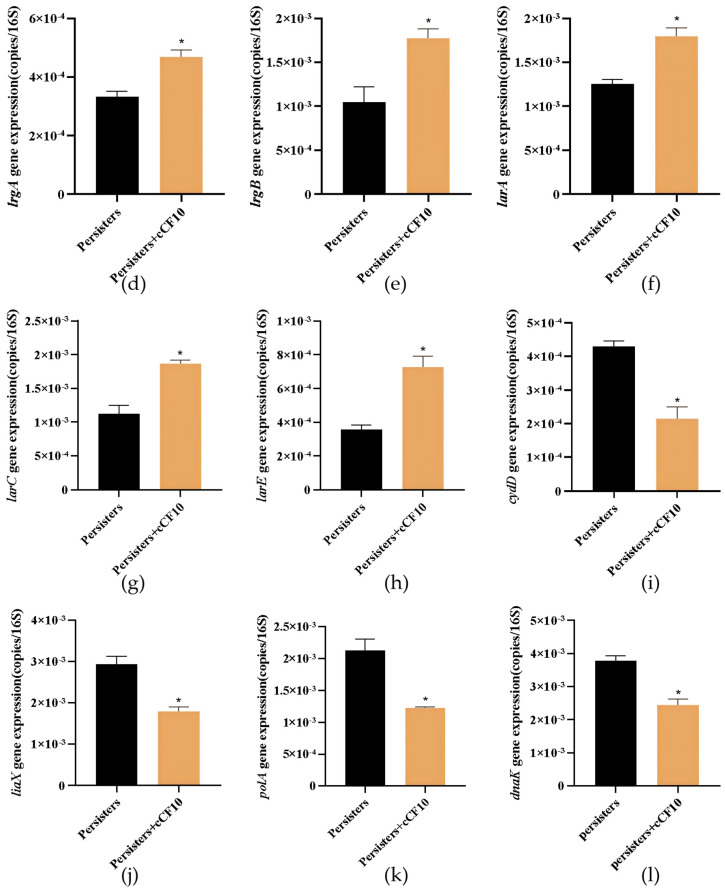
Transcriptomic profiling of cCF10-treated *E. faecalis* OG1RF (pCF10) persister cells and qRT-PCR validation of key genes. (**a**) Volcano plot of DEGs between the untreated persister group and cCF10-treated persister group. (**b**) Bubble plot of GO functional enrichment analysis for DEGs. (**c**) Bubble plot of KEGG pathway enrichment analysis for DEGs. (**d**–**l**) Relative expression levels of key genes related to persister formation. The results represent the mean ± SD of three biological replicates. Significant differences between groups are presented with * *p* < 0.05.

Transcriptomic sequencing was performed on *E. faecalis* OG1RF (pCF10) persister cells with or without 10 μg/L cCF10 pretreatment, using the same DEG significance threshold of |log_2_ (fold change)| > 1 and *p*-value < 0.05. Volcano plot analysis of differentially expressed genes (DEGs) identified a total of 162 DEGs, which suggested that cCF10 mediated more extensive state-specific transcriptomic remodeling in persister cells. Functional enrichment analysis revealed that these DEGs were primarily involved in biological processes including cell wall synthesis and remodeling, transmembrane transport, amino acid metabolism, quorum sensing, and stress response regulation. These DEGs were enriched in pathways closely related to bacterial stress resistance and dormancy maintenance, including quorum sensing, phosphotransferase systems, and arginine biosynthesis, which provided transcriptomic-level support for the enhancement of the persisting phenotype (Figure 3b,c).

Gene expression analysis revealed that cCF10 treatment upregulated the expression of *lrgA* and *lrgB*—genes associated with peptidoglycan synthesis and cell wall homeostasis regulation—by 1.40-fold and 1.69-fold (Figure 3d,e), respectively, and simultaneously synergistically upregulated the transcriptional levels of the *lar* gene cluster (*larA*, *larC*, *larE*) associated with lactic acid racemization (Figure 3f–h). These transcriptional changes suggest a potential role for cCF10 in enhancing cell wall stability, which may contribute to improved bacterial survival under antibiotic stress. Analysis of stress response- and energy metabolism-related gene expression showed that cCF10 downregulated the expression of genes associated with high-energy-consuming stress pathways in persister cells, including the ABC transporter gene *cydD* (encoding cytochrome bd oxidase assembly-related proteins) (Figure 3i), the membrane protein gene *liaX* (encoding cell envelope stress sensors) (Figure 3j), the DNA polymerase I gene *polA* (encoding DNA repair-related enzymes) (Figure 3k), and the Hsp70 family chaperone gene *dnaK* (Figure 3l). The coordinated downregulation of these energy-intensive genes forms a transcriptional pattern that matches the known characteristics of metabolically quiescent persister cells, which may contribute to the long-term survival of this bacterial subpopulation under sustained antibiotic exposure.

These results indicate that cCF10 enhances the stability and antibiotic resistance of persister cells via a bidirectional regulatory strategy that drives transcriptional reprogramming in these cells, thereby strengthening cellular structural resistance and suppressing energy metabolism.

### 3.3. cCF10-Mediated Downregulation of Its Own Pheromone Sensing and Uptake System

**Figure 4:**
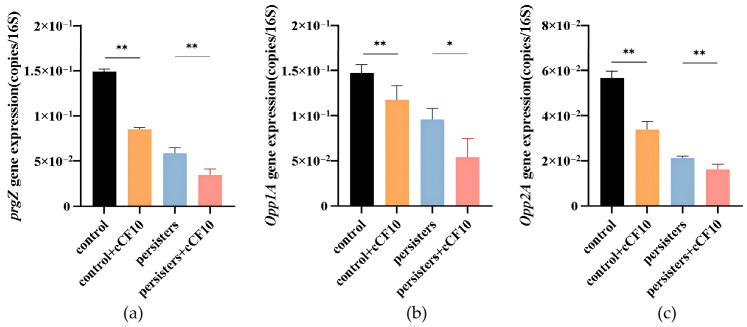
Expression levels of cCF10 uptake-related genes across experimental groups. (**a**) *prgZ*. (**b**) *Opp1A*. (**c**) *Opp2A*. The results represent the mean ± SD of three biological replicates. Significant differences between groups are presented with * *p* < 0.05 and ** *p* < 0.01.

Gene expression analysis revealed that the pheromone-sensing- and uptake-related genes *prgZ*, *opp1A,* and *opp2A* were downregulated in cCF10-treated normal bacteria, untreated persister cells, and cCF10-pretreated persister cells, with the lowest expression of all three genes detected in the cCF10-pretreated persister group (Figure 4).

prgZ, encoded by the pCF10 plasmid, encodes the cCF10 pheromone-binding protein, which cooperates with the Opp1 and Opp2 oligopeptide permease systems to mediate cCF10 recognition and intracellular uptake. The synergistic downregulation of these genes indicates broad inhibition of the cCF10 sensing pathway in persister cells. As the expression of ABC transport systems (such as the Opp system) and substrate transport processes is highly energy-consuming, this inhibition aligned with the energy conservation strategy of dormant persister cells.

### 3.4. Activation of the Stringent Response Pathway by cCF10 in E. faecalis Persister Cells

**Figure 5:**
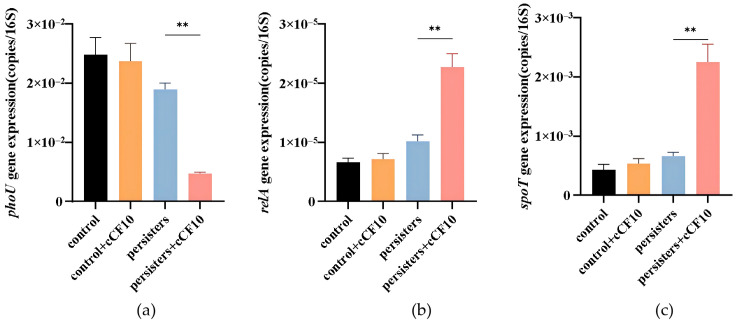
Expression levels of stringent response pathway-related genes in each experimental group. (**a**) *relA*. (**b**) *spoT*. (**c**) *phoU*. The results represent the mean ± SD of three biological replicates. Significant differences between groups are presented with ** *p* < 0.01.

The bacterial stringent response is a core regulatory hub for adaptation to nutrient deprivation and environmental stress. Gene expression analysis revealed that cCF10 upregulated the expression of *relA* and *spoT*, the core genes of the stringent response, during *E. faecalis* persister formation, while synergistically downregulating the transcription of *phoU*, a global metabolic regulator (Figure 5). The expression of *relA* and *spoT* peaked in cCF10-treated persister cells, indicating that cCF10 strongly activated the (p)ppGpp-mediated stringent response signaling pathway. Meanwhile, *phoU* expression was lowest in the cCF10-pretreated persister group, consistent with the established regulatory link between *phoU* repression and intracellular (p)ppGpp accumulation. These results demonstrate that cCF10 modulates the stringent response and global metabolic programming to promote the formation and maintenance of *E. faecalis* persister cells.

## 4. Discussion

Bacterial persistence is a non-heritable phenotype characterized by high antibiotic tolerance, which contributes to recurrent *E. faecalis* infections and antimicrobial treatment failure and provides an evolutionary reservoir for the acquisition of heritable resistance [3,8,18]. However, the role of this signaling axis in persister formation has remained unclear.

**Figure 6:**
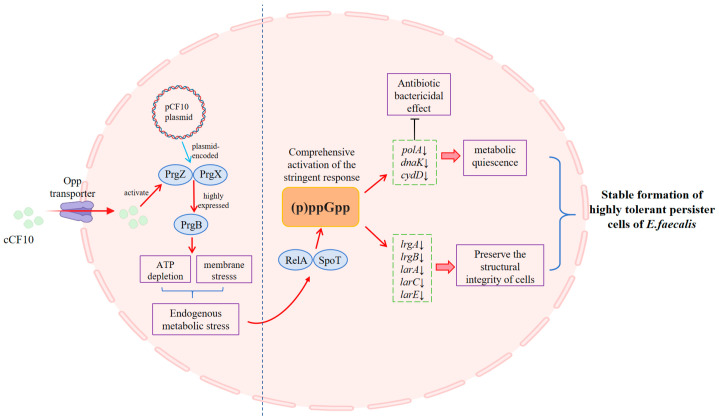
Molecular regulation mechanism diagram of cCF10-mediated persistence of *E. faecalis* OG1RF (pCF10).

In this study, we first optimized and established a persister screening model for *E. faecalis* OG1RF (pCF10) using LVFX. Based on this model, we systematically elucidated the molecular mechanism by which cCF10 promotes the formation of a high-tolerance persister phenotype in *E. faecalis* via cascade transcriptional regulation and global transcriptomic remodeling (Figure 6). This work reveals the core logic of phenotypic switching driven by the adaptive cost of conjugative plasmids in pathogenic bacteria and provides a novel theoretical basis and potential intervention targets for the prevention and control of persistent infections caused by clinically multidrug-resistant *E. faecalis*.

This study found that the regulatory effect of cCF10 on the persister phenotype of *E. faecalis* was strictly dependent on the pCF10 plasmid. In the OG1RF strain carrying the pCF10 plasmid, cCF10 increased the persister frequency of the strain to LVFX in a concentration-dependent manner, with the maximum increase from 0.291% in the control group to 16.466%, a 56-fold elevation. Growth curve results confirmed that cCF10 had no significant effect on the growth rate of the wild-type OG1RF strain without the pCF10 plasmid and only exerted a physiological proliferation-slowing effect on the plasmid-carrying strain, further verifying the plasmid specificity of this regulatory effect.

The core cause of this phenotypic reversal lies in the cCF10-specific response system conferred to the strain by the pCF10 plasmid, as well as the endogenous physiological stress induced by the conjugation process, which also constitutes the initial link of the entire regulatory pathway. Extracellular cCF10 first completes transmembrane transport into the cell via the Opp oligopeptide transport system on the bacterial membrane [30,31,32] and then binds to the specific receptors PrgZ and PrgX encoded by the pCF10 plasmid, releasing transcriptional repression of the docking operon and driving explosive expression of structural proteins such as PrgB and other binding devices. This process imposes a substantial metabolic burden on cells, which aligns with the reported theory that metabolic restraint is associated with bacterial persister formation [20,21].

The endogenous metabolic stress signal induced by cCF10 ultimately drives the core transition from stress signal to persister phenotype by activating the stringent response mediated by (p)ppGpp. The stringent response also acts as the central hub of the entire regulatory pathway, linking upstream stress signals to downstream persister-promoting effects. Gene expression analysis in this study showed that cCF10 treatment upregulated the expression of the stringent response core genes *relA* and *spoT* in persister cells. *relA* and *spoT* are responsible for the synthesis and bidirectional regulation of (p)ppGpp, respectively, and are the core executive genes of the stringent response in *E. faecalis* [33]. The two genes synergistically drove intracellular (p)ppGpp accumulation, fully activating the stringent response signaling axis.

The stringent response after activation further drove the reconstruction of the global transcriptome: on the one hand, it systematically inhibited the expression of high-energy non-essential pathways, including the DNA repair-related *polA* [34], protein misfolding repair-related *dnaK* [35], and respiratory chain assembly-related *cydD* [36,37], forcing cells to shut down proliferation-related biosynthesis and repair processes and enter a deep metabolic resting state. This is also the core molecular basis for persister cells to achieve high antibiotic tolerance. On the other hand, cCF10 synergistically upregulated the expression of anti-autolysis-related genes *lrgA* and *lrgB* [38], as well as lactate racemization system-related genes *larA*, *larC*, and *larE* [39,40]. The lrgAB system maintains cell wall integrity and resists antibiotic-induced cell lysis by inhibiting autolysin activity, while the *lar* gene cluster reshapes cell wall peptidoglycan by mediating lactate racemization to enhance the structural stability of the cell wall. The synergistic high expression of these two systems provides a structural survival guarantee for host bacteria in a metabolic dormant state.

On the basis of stringent response-driven metabolic dormancy and enhanced structural defense, cCF10 also induced a negative feedback desensitization mechanism of the pheromone sensing system, forming a closed regulatory loop for the entire pathway. Gene expression analysis in this study revealed that the pheromone receptor-encoding gene *prgZ* and the Opp oligopeptide transport system genes that mediate cCF10 intracellular uptake were downregulated in cCF10-pretreated persister cells. This downregulation may reduce energy consumption related to signal transport and help stabilize the quiescent state of persister cells.

In addition, during the pre-adaptation stage prior to persister phenotype establishment, cCF10 upregulated the expression of the alcohol dehydrogenase-encoding gene adhE and the ATP synthase subunit-encoding gene atpH during the active growth phase. This upregulation enhanced the intracellular fermentation pathway and energy synthesis, completed the pre-stress energy reserve, and laid a metabolic foundation for the subsequent establishment of the persister state [41]. Meanwhile, cCF10 upregulated the biofilm core pilus subunit-encoding genes *ebpA* and *ebpB*, enhanced bacterial surface adhesion and intercellular aggregation, and built a physical barrier against antibiotic penetration and host immune clearance for the persister subpopulation [42,43].

However, all experiments in this study were conducted in vitro using a single laboratory strain of *E. faecalis* OG1RF (pCF10). Further validation in clinically relevant in vivo infection models, as well as across genetically diverse clinical multidrug-resistant *E. faecalis* isolates, is essential before these observations can inform the rational design of clinical anti-persister strategies.

In summary, this study systematically elucidated the cascade regulatory mechanism of cCF10-mediated persister formation in *E. faecalis* OG1RF (pCF10). We revealed that metabolic restraint and stringent response activation triggered by pCF10-dependent signaling are key drivers of persister formation. These findings expand the current understanding of the crosstalk between quorum sensing, conjugative plasmids, and bacterial persistence and provide a theoretical foundation for the development of targeted anti-persister therapies against multidrug-resistant *E. faecalis*.

## 5. Conclusions

This study systematically elucidated the molecular mechanism by which the pheromone cCF10 regulates persister formation in *E. faecalis* harboring the pCF10 conjugative plasmid. The results demonstrated that cCF10 induced comprehensive transcriptional reprogramming centered on the activation of the stringent response and the downregulation of energy-intensive metabolic pathways, thereby establishing a mechanistic framework linking plasmid-encoded signaling systems to phenotypic antibiotic tolerance. While these findings suggest potential anti-persister targets, further validation in clinically relevant models and diverse clinical isolates is required. This work expands the cognitive boundaries of cross-regulation between bacterial quorum sensing and persister phenotype formation and provides a theoretical foundation for future studies aimed at preventing persistent *E. faecalis* infections.

## Data Availability

The original contributions presented in this study are included in the article/Supplementary Material. Further inquiries can be directed to the corresponding authors.

## Supplementary Materials

The following supporting information can be downloaded at https://www.mdpi.com/article/10.3390/microorganisms14050960/s1. Figure S1: Standard curve of target genes; Figure S2: MIC of LVFX against Enterococcus faecalis OG1RF (pCF10); Figure S3: Validation of Antibiotic Sensitivity of Resuscitation Bacteria. Table S1: Sequence of primers for qRT-PCR analysis. Text S1: Transcriptome sequencing and data analysis.
