# Supplementary material for: Pheromone cCF10 Enhances Persister Formation in Enterococcus faecalis via Transcriptomic Changes
Source: Microorganisms. 2026 Apr 24;14(5):960. doi: 10.3390/microorganisms14050960 (PMC13210022; doi:10.3390/microorganisms14050960)
Supplement: Supplementary file 1 [file microorganisms-14-00960-s001.zip › Supplementary materials.pdf]

Article

Pheromone cCF10 Enhances Persister Formation in *Enterococcus faecalis* via Transcriptomic Changes

Jingxue Qian [1,2,†], Xiaobo Yang [2,†], Rumeng Li [2,3], Man Zhang [2,4], Ruolin Hao [2], Qing He [2], Lin Xu [2,3], Zhiqiang Shen [2], Jingfeng Wang [2], Feilong Sun [1,*] and Zhigang Qiu [2,*]

[1] College of Environmental and Chemical Engineering, Xi'an Polytechnic University, Xi'an 710600, China
[2] Military Medical Sciences Academy, Academy of Military Sciences, Tianjin 300050, China
[3] College of Oceanography and Ecological Science, Shanghai Ocean University, Shanghai 201306, China
[4] The Third Central Clinical College of Tianjin Medical University, Tianjin 300170, China
* Correspondence: sunfeilong@xpu.edu.cn (F.S.); zhigangqiu99@gmail.com (Z.Q.)
† These authors contributed equally to this work.

1 Supplementary Texts

Text S1 Transcriptome sequencing and data analysis

Experimental method

Total RNA was isolated using the Trizol Reagent (Invitrogen Life Technologies). Quality and integrity were determined using a NanoDrop spectrophotometer (Thermo Scientific) and a Bioanalyzer 2100 system (Agilent). Zymo-Seq RiboFree Total RNA Library Kit was used to remove rRNA from total RNA. Random oligonucleotides and SuperScript III were used to synthesize the first strand cDNA. Second strand cDNA synthesis was subsequently performed using DNA Polymerase I and RNase H (Strand-specific RNA-seq：Then use RNaseH to degrade the RNA strand, and in the DNA polymerase I system, use dNTP with dUTP instead of dTTP as raw material to synthesize the second strand of cDNA). Remaining overhangs were converted into blunt ends via exonuclease/polymerase activities and the enzymes were removed. After adenylation of the 3′ends of the DNA fragments, Illumina PE adapter oligonucleotides were ligated to prepare for hybridization. To select cDNA fragments of the preferred 400-500 bp in length, the library fragments were purified using the AMPure XP system (Beckman Coulter, Beverly, CA, USA). DNA fragments with ligated adaptor molecules on both ends were selectively enriched using Illumina PCR Primer Cocktail in a 15 cycle PCR reaction. Products were purified (AMPure XP system) and quantified using the

Agilent high sensitivity DNA assay on a Bioanalyzer 2100 system (Agilent). The sequencing library was then sequenced on NovaSeq 6000 platform (Illumina) by Shanghai Personal Biotechnology Cp. Ltd.

Transcriptome analysis flow

1) Data quality control

The quality information of raw data in FASTQ format was calculated and then the raw data was filtered using fastp (0.22.0) software, clean data was obtained by removing reads containing adapter, reads containing ploy-N and low quality reads. All the subsequent analysis was based on high quality clean data .

2) Data mapping analysis

The reference genome and gene annotation files were downloaded from genome website. Reference genome index was built by Bowtie2 (2.4.1) and the filtered reads were mapping to the reference genome using Bowtie2.

3)Expression level analysis

The gene read count value was counted using HTSeq (v0.9.1) as the original expression level of the gene. In order to make the gene expression levels of different genes and different samples comparable, FPKM(fragments per kilobaseof exon per million fragments mapped) is used to normalize the expression.

4) Differential expression analysis

Then difference expression of genes was analyzed by DESeq2 (v1.38.3) with screened conditions as follows: expression difference multiple |log2FoldChange| > 1, significant P-value < 0.05. At the same time, we used ComplexHeatmap (v2.16.0) software package to perform bi-directional clustering analysis of all different genes of samples. We geted heatmap according to the expression level of the same gene in different samples and the expression patterns of different genes in the same sample with Euclidean method to calculate the distance and Complete Linkage method to cluster.

5) Enrichment analysis:

We mapped all the genes to Terms in the Gene Ontology database and calculated the numbers of differentially enriched genes in each Term. Using ClusterProfiler (v4.6.0) to perform GO enrichment analysis on the differential genes (all DEGs / up DEGs / down DEGs), calculate P-value by hypergeometric distribution method (the standard of significant enrichment is P-value <0.05), and find the GO term with significantly enriched differential genes to determine the main biological functions performed by differential genes.

ClusterProfiler (v4.6.0) software was used to carry out the enrichment analysis of the KEGG pathway of differential genes, focusing on the significant enrichment pathway with P-value <0.05.

6)GSEA (Gene Set EnrichmentAnalysis)

ClusterProfiler (v4.6.0) tool was used for GSEA enrichment analysis of all genes, and GSEA enrichment analysis pathway map was drawn.

7) New transcript analysis（Strand-specific RNA-seq）

The transcriptome data were analyzed by specifying the reference genome by Rockhopper (v2.03) software to obtain the transcript regions on the genome without transcript annotation. The sequence of this region compared with known databases for BLASTX.

8) sRNA（Strand-specific RNA-seq）

Transcripts without any known sequence on the match will be subjected to further sRNA analysis. Small (~30-500 nt) non-coding RNAs (non-coding RNAs, sRNAs) in bacteria are the most abundant species of post-transcriptional regulators. Transcripts without any known sequence on the match were predicted by RNAfold (2.4.6) for secondary structure .

Rfam database was used to annotate sRNA, and RNAplex were used to predict sRNA target genes.

9) AntisensTranscript（Strand-specific RNA-seq）

Cis-natural antisense transcripts (cis-NATs) are endogenous RNA molecules transcribed from the same region of the source DNA strand as the righteous transcript with partially convergent or dispersed directional repeats. There are three types of antisense transcripts: enclosed, convergent and divergent. For strand-specific library RNA-seq data, the location, type and number of antisense transcripts on the genome can be identified.

10) UTR

For the 5' UTR, the 25 bp upstream sequence of the predicted gene was analyzed by ELFH(v1.0.1) software to find possible RBS sequences. For the 3' UTR, the sigma-independent terminator was predicted using TransTermHP(v2.08) software.

11) Operon

Transcript Start Site (TSS) and Transcription Termination Site (TTS) were predicted by Rockhopper(v2.03)

12) SNP and InDel analysis

The Varscan (v2.3.9) software was used to obtain SNP and InDel sites, and the filtering criteria were:

1)SNP site base Q>20;

2)The number of Reads covering the site> 8;

3)The number of Reads supporting the mutation site> 2;

4)The p-value of SNP locus is <0.01. Analysis of variant sites.

The data were analyzed by using the free online platform Personalbio GenesCloud.

2 Supplementary Figures and Tables

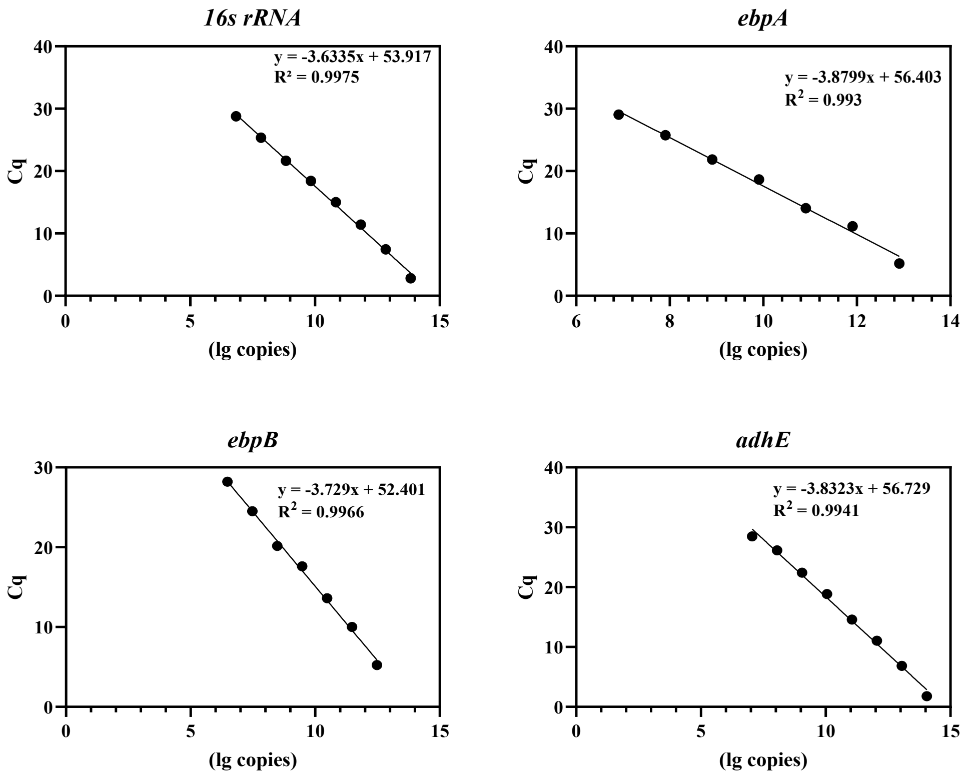

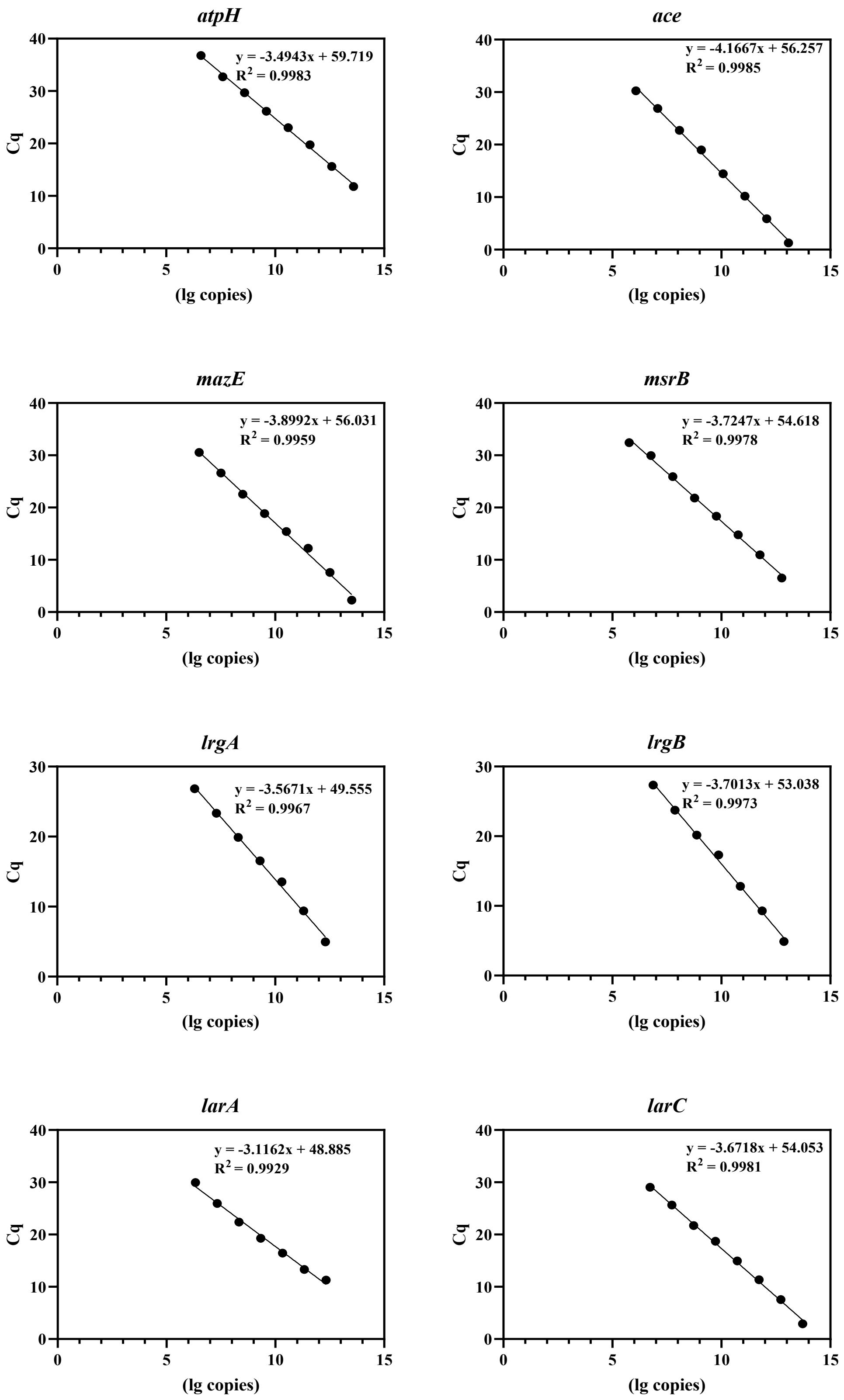
atpH
y = -3.4943x + 59.719
R2 = 0.9983
ace
y = -4.1667x + 56.257
R2 = 0.9985
mazE
y = -3.8992x + 56.031
R2 = 0.9959
msrB
y = -3.7247x + 54.618
R2 = 0.9978
lrgA
y = -3.5671x + 49.555
R2 = 0.9967
lrgB
y = -3.7013x + 53.038
R2 = 0.9973
larA
y = -3.1162x + 48.885
R2 = 0.9929
larC
y = -3.6718x + 54.053
R2 = 0.9981
Cq
(lg copies)
0
5
10
15
20
30
40

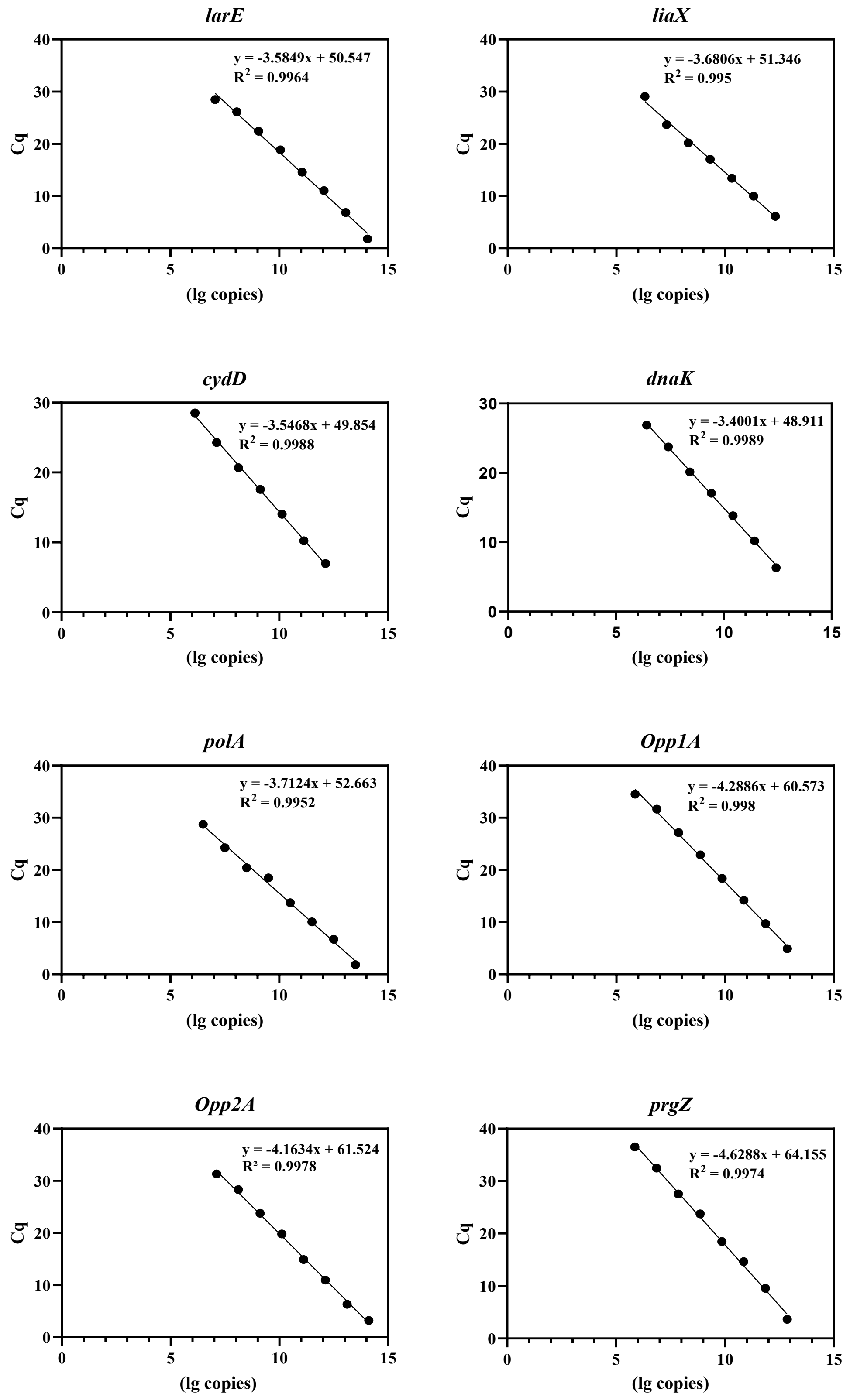
larE
y = -3.5849x + 50.547
R^2 = 0.9964
Cq
(lg copies)
liaX
y = -3.6806x + 51.346
R^2 = 0.995
Cq
(lg copies)
cydD
y = -3.5468x + 49.854
R^2 = 0.9988
Cq
(lg copies)
dnaK
y = -3.4001x + 48.911
R^2 = 0.9989
Cq
(lg copies)
polA
y = -3.7124x + 52.663
R^2 = 0.9952
Cq
(lg copies)
Opp1A
y = -4.2886x + 60.573
R^2 = 0.998
Cq
(lg copies)
Opp2A
y = -4.1634x + 61.524
R^2 = 0.9978
Cq
(lg copies)
prgZ
y = -4.6288x + 64.155
R^2 = 0.9974
Cq
(lg copies)

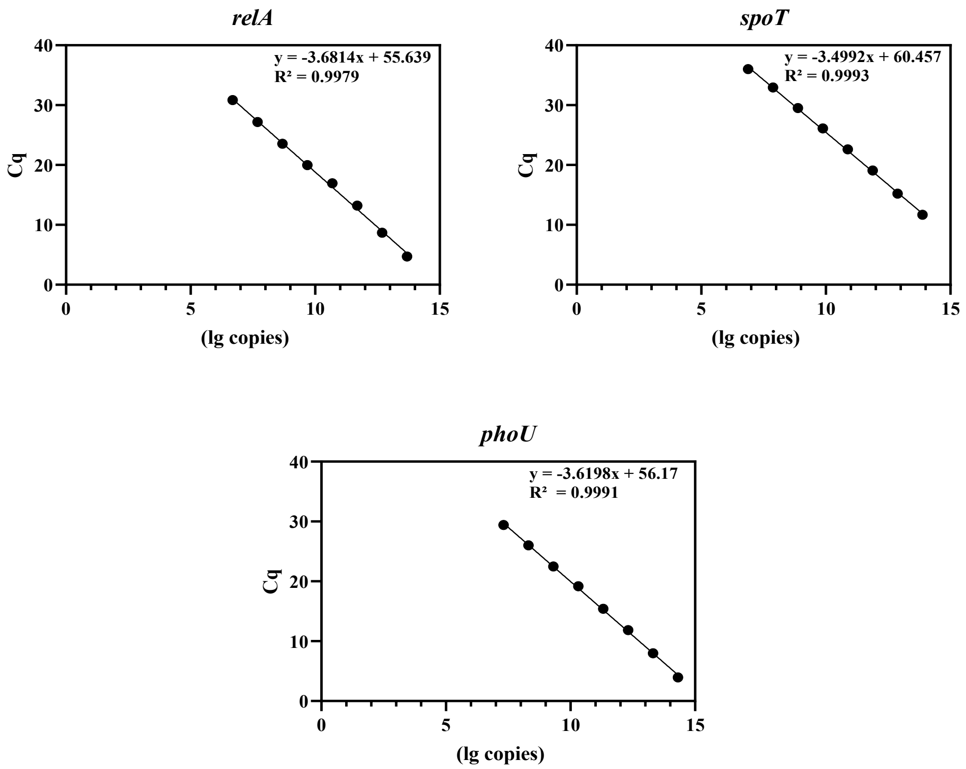

Figure S1. Standard curve of target genes

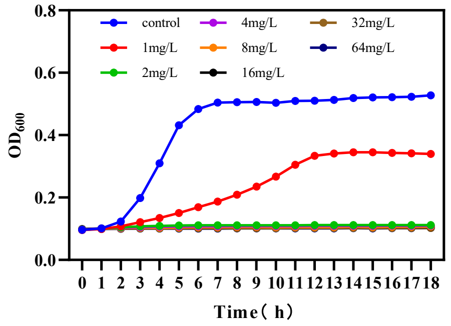

Figure S2. MIC of LVFX against Enterococcus faecalis OG1RF (pCF10)

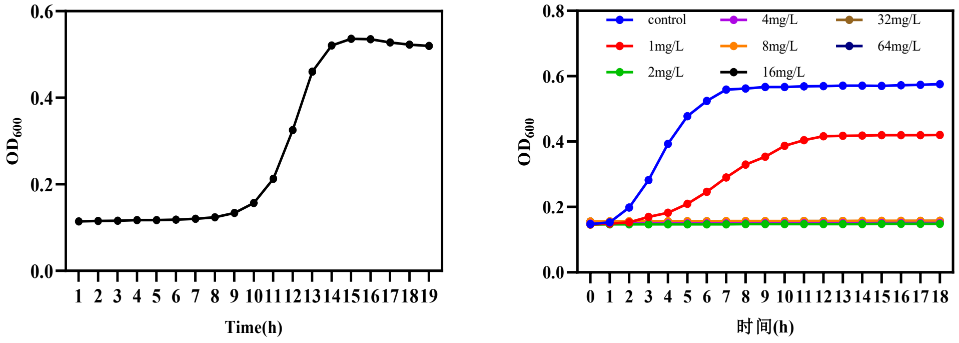

(a) (b)

Figure S3. (a)Recovery curve of OG1RF (pCF10) retained bacteria
(b)MIC of LVFX on OG1RF (pCF10) Resuscitation Strains

Table S1 Sequence of primers for qRT-PCR analysis

Gene name	Primer sequences (5 'to 3')	Product length (BP)
ebpA	F: AGACGGTAGTGCACAATGGG<br>R: TGGTCTCCTGTACCGCCATA	96
ebpB	F: GCTACTCGCTCTTTTCGGGT<br>R: CTTCCCCTGTGTTTTGCTGC	131
adhE	F: AGAAGCAGCACGAGTGGTAC<br>R:TTGACCATTCCAGCTCCACC	159
atpH	F: GCAACTGGACGAAAACGGTC<br>R: AACTGCTCCAGAACATCCGG	87
mazE	F:CGGAAAGAAACCTTCAGAAAACCA<br>R:AATACTCGGCCTCTTCCCCA	116
ace	F:GAGGCAAGCGGCAATCAAAA<br>R:TTTCTCTTGCGCCGTTCTCT	283
lrgA	F:AACGGCTTGGCGTTCATTTC<br>R:CTAGCCCTTCCACTTGCTCC	115
lrgB	F:TGGTTCGCTAGTGGCTGTTT<br>R:CCGTAATGGAAGCAATGCCG	143
larA	F:ACGAATGGCGGATTTCCACT<br>R:ACACCGCCGTCTTTATTGGT	89
larC	F:GCTTGACTTAGGCGGGGATT	85

	R:TCGTTGGGCTTGAATGGTGA	
larE	F:GTTCTGGCCGATGGGATGAT<br>R:TAGCGGACTGATGACCCCTT	90
cydD	F:GTTGGCTTAGACGAGCTGGT<br>R:TTTTGATCGACAAAGGCGCG	128
polA	F:ATGATTGAAATGGCGCGTCG<br>R:TGCGTGCTCCATCACTTCTT	144
liaX	F:TGCGTGCTCCATCACTTCTT<br>R:CAGAGCTTGCTTCAACACGG	108
danK	F:CGGTGAAATTCAAGTGGGCG<br>R:GTAGCCAGCTTCACCCATGT	97
Opp1A	F:AGACGGCTTTGATGGTCCTG<br>R:TCAACGCGGTTGGTGATTCT	131
Opp2A	F:GGCGGTGTTTGGGGTTCAGT<br>R:TTTCGCTTTCATCGCTTCTACAAT	249
prgZ	F:GACTTTGACTGCAGGGACACC<br>R:AGCGCCATCTCTAATCACAAT	251
relA	F:TGAACGGGCAATGCAGGAAGAGT<br>R:AACGCCACCGACGCCAATCACAAA	319
spoT	F:GCCGCATCGCCCGTGAAACTCT<br>R:TGGCCCAGCACGCGATAACAGGT	370
phoU	F:GGTTAGTAGTGCCGTCCAT<br>R: GGTCCGCCATTCTTTCTA	221
16S RNA	F:GCGGCGTGCCTAATACA<br>R:CCGCGGGTCCATC	219
